# Study on the Effect of Bee Venom and Its Main Component Melittin in Delaying Skin Aging in Mice

**DOI:** 10.3390/ijms26020742

**Published:** 2025-01-16

**Authors:** Huiting Zhao, Miaomiao Liu, Longlong Chen, Yu Gong, Weihua Ma, Yusuo Jiang

**Affiliations:** 1 College of Life Science, Shanxi Agricultural University, Taigu 030801, China; zhaohuiting@sxau.edu.cn (H.Z.);; 2 College of Horticulture, Shanxi Agricultural University, Taiyuan 030031, China; mawh1997@163.com; 3 College of Animal Science, Shanxi Agricultural University, Taigu 030801, China

**Keywords:** bee venom, skin aging, melittin, collagen, metabolomics

## Abstract

Bee venom (BV) and its main compound melittin (MLT) have antioxidant, anti-inflammatory, and anti-aging activities; however, very little research has been conducted on their effects on skin aging. In this study, a mouse skin aging model induced by D-galactose was constructed via subcutaneous injection into the scruff of the neck, and different doses of BV and MLT were used as interventions. The anti-aging effects and mechanisms of BV and MLT were explored by detecting the skin morphology and structure, and anti-aging-related factors and performing non-targeted metabolomics of mice. BV and MLT improved dermal and epidermal thickness, boosted the collagen fiber content, increased hydroxyproline and hyaluronic acid levels, and enhanced transcript-level expression of *IL-10*, *Col1a1*, and *Col3a1*, while decreasing that of *IL-1β*. Metabolomic analysis showed that BV and MLT regulated the levels of some metabolites (compared to those in the skin aging control). BV effectively alleviated skin aging by regulating the pentose phosphate pathway, and pathways associated with carbon, galactose, and β-alanine metabolism, whereas MLT regulated pathways related to lipid metabolism, cholesterol metabolism, and atherosclerosis. This study highlights the potential applicability of BV and MLT in skin aging treatments and cosmetic products.

## 1. Introduction

Aging involves the physical degeneration of the body over time [1]. As a barrier organ with the largest contact area between the body and external environment, the skin displays key manifestations indicative of organismal aging [2]. Skin aging is caused by intrinsic and extrinsic factors. Endogenous aging is an unavoidable process that manifests as wrinkles, skin dryness, laxity, and loss of elasticity [3]. Intrinsic factors that drive skin aging include age, genetics, growth factors, and hormonal changes [4]. Exogenous factors include ultraviolet rays, air pollution, cigarette smoke, and stress, which frequently result in skin roughness, hyperpigmentation, and the appearance of age spots [5].

Skin aging not only affects esthetics but also increases the risk of infections, chronic wounds, and various types of dermatitis and malignant tumors, including melanoma [6]. With improvement in the quality of living standards and development in science and technology, products that prevent and treat skin aging have become popular commodities. However, many current anti-aging drugs have certain limitations, such as low safety and bioavailability [7]. Therefore, exploring the mechanisms underlying skin aging is essential for facilitating the development of safe, effective, and economical anti-aging drugs.

Bee venom (BV) is an odorless, transparent, acidic liquid secreted by the venomous or paracrine glands of worker bees; compositionally, it contains a variety of active molecules, including peptide and non-peptide components, among which melittin (MLT) is the primary component [8,9]. Recently, the cosmetic applications of BV, for example, its use as an anti-wrinkle agent, have garnered significant attention. Additionally, BV has been shown to be effective at reducing the total number of wrinkles and wrinkle area in photodamaged skin [10]. Furthermore, because of the low-irritant-potential of BV, the long-term usage of cosmetics containing BV is considered safe [11]. BV exerts an inhibitory effect on the expression of ultraviolet-induced matrix metalloproteinases MMP-1 and MMP-3 in human skin fibroblasts. The use of BV as a photoprotective agent against ultraviolet B has been reported in [12]. However, the mechanisms underlying the anti-aging effects of BV are yet to be elucidated comprehensively are limited.

In this study, we used D-galactose (D-gal) to generate a mouse model of aging and systematically analyzed the anti-skin aging effects of BV and its main component, MLT, by observing skin tissue sections. We also detected the expression of anti-aging factors, collagen, and inflammatory factors and performed metabolomic analysis to compare the differences before and after anti-aging treatments. We hope this study will lay a theoretical foundation for the pharmacological effects of BV and its application in the cosmetic and skin care industries.

## 2. Results

### 2.1. Effects of BV and MLT on Skin Morphology

**Figure 1:**
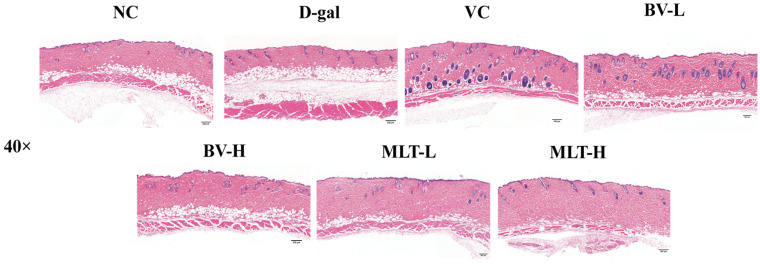
Hematoxylin-eosin staining images of skin tissue of mice. NC, normal control; D-gal, D-galactose; VC, positive control; BV-L, low dose of bee venom (0.1 mg·kg^−1^); BV-H, high dose of bee venom (1 mg·kg^−1^); MLT-L, low dose of melittin (0.05 mg·kg^−1^); and MLT-H, high dose of melittin (0.5 mg·kg^−1^). Scale bar = 200 μm.

Hematoxylin-eosin (HE) staining showed that tissue sections from the normal control (NC) group had complete epidermis and dermis, tight epidermal–dermal connections, thicker dermis, an orderly arrangement of dermal fibrous tissues, and fewer subcutaneous adipocytes (Figure 1). The epidermis and dermis became thinner, and the connections between the epidermis and dermis were not tight, with disordered arrangements of the fibrous tissues of the dermis and an increased number of subcutaneous adipocytes in the D-gal-induced skin-aging group. The positive control Vitamin C (VC) group displayed increased epidermal and dermal thickness, more hair follicles, and more regular epidermal and dermal tissues. Both high and low doses of BV (BV-H and BV-L) and MLT (MLT-H and MLT-L) treatments in mice with D-gal-induced aging altered the morphology of skin tissues. The epidermal and dermal layers were thicker and exhibited a more regular structure, with a well-defined and tighter epidermis–dermis junction. The fibrous tissues in the dermis were more uniformly arranged, with a noticeable reduction in subcutaneous adipocytes.

**Figure 2:**
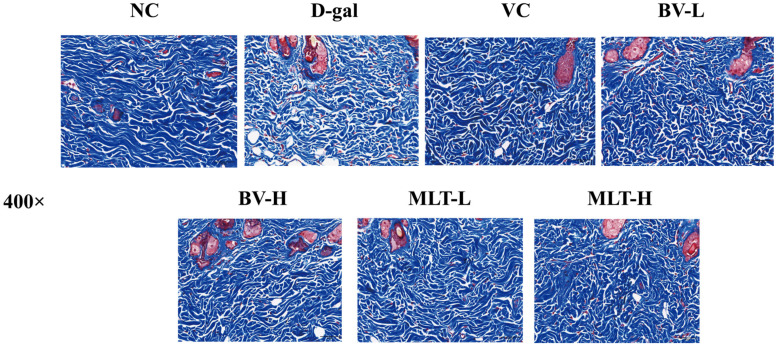
Masson trichrome staining images of skin tissue of mice. NC, normal control; D-gal, D-galactose; VC, positive control; BV-L, low dose of bee venom (0.1 mg·kg^−1^); BV-H, high dose of bee venom (1 mg·kg^−1^); MLT-L, low dose of melittin (0.05 mg·kg^−1^); and MLT-H, high dose of melittin (0.5 mg·kg^−1^). Scale bar = 50 μm.

Masson trichrome staining of NC group skin sections highlighted neatly and tightly arranged collagen fibers in bright blue color. Collagen fibers in the D-gal group were stained in a lighter shade of blue; they were significantly reduced in length, partially broken, and loosely arranged. In the VC, BV-L, BV-H, MLT-L, and MLT-H groups, collagen fibers in the dermis were regular, more abundant, and tightly arranged (Figure 2).

### 2.2. Effects of BV and MLT on Hyaluronic Acid and Hydroxyproline Content

**Figure 3:**
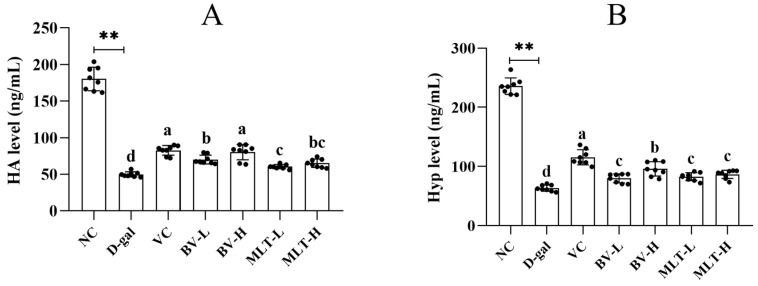
Effect of BV and MLT on the hydroxyproline (**A**) and hyaluronic acid (**B**) content in mouse skin sections. NC, normal control; D-gal, D-galactose; VC, positive control; BV-L, low dose of bee venom (0.1 mg·kg^−1^); BV-H, high dose of bee venom (1 mg·kg^−1^); MLT-L, low dose of melittin (0.05 mg·kg^−1^); and MLT-H, high dose of melittin (0.5 mg·kg^−1^). ** indicates *p* < 0.01 vs. NC. Different letters indicate *p* < 0.05 between treatment groups.

The hyaluronic acid (HA) and hydroxyproline (Hyp) content in the D-gal group were significantly decreased compared with that in the NC group (*p* < 0.05), suggesting that aging reduces the content of HA and Hyp in the skin. The BV- and MLT-treated groups showed a significantly higher content of HA and Hyp than the D-gal group (*p* < 0.05). The VC and BV-H groups had significantly higher levels of HA than the other treatment groups (*p* < 0.05). However, the difference between the BV-L and MLT-H groups was not significant, nor was there a difference between the MLT-H and MLT-L groups (Figure 3A). The VC group had the highest levels of Hyp, significantly higher than those of the other treatment groups (*p* < 0.05), followed by the BV-H group, in which the levels were significantly higher than those in the remaining three groups (BV-L, MLT-H, and MLT-L); there was no significant difference among these three groups (Figure 3B). These results indicated that BV and MLT promote the production of HA and Hyp in mouse skin, with high doses of BV having the strongest effect.

### 2.3. Effects of BV and MLT on the Expression of Collagen and Inflammatory Factors

**Figure 4:**
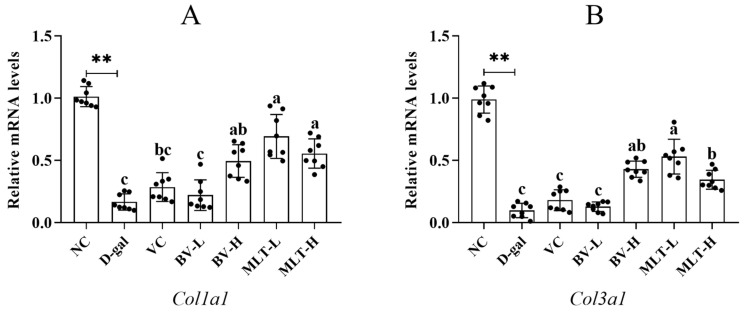
Effect of BV and MLT on the expression of *Col1a1* (**A**) and *Col3a1* (**B**) in mice skin. NC, normal control; D-gal, D-galactose; VC, positive control; BV-L, low dose of bee venom (0.1 mg·kg^−1^); BV-H, high dose of bee venom (1 mg·kg^−1^); MLT-L, low dose of melittin (0.05 mg·kg^−1^); and MLT-H, high dose of melittin (0.5 mg·kg^−1^). ** indicates *p* < 0.01 vs. NC. Different letters indicate *p* < 0.05 between treatment groups.

The expression levels of *Col1a1* and *Col3a1* in the D-gal group were significantly decreased compared with those in the NC group (*p* < 0.01). Compared with those in the D-gal group, the expression levels of *Col1a1* and *Col3a1* were elevated in all BV and MLT treatment groups, and the expression pattern was similar for both genes (Figure 4). The BV-H, MLT-H, and MLT-L groups had significantly higher expression levels than those of the D-gal group (*p* < 0.05), with the MLT-L group showing the highest levels. There were no significant differences among the VC, BV-L, and D-gal groups. This indicates that BV-H, MLT-L, and MLT-H effectively increase the expression levels of genes related to collagen synthesis in the mouse skin and improve the loss of skin collagen owing to aging, with low-dose melittin having the strongest effect.

**Figure 5:**
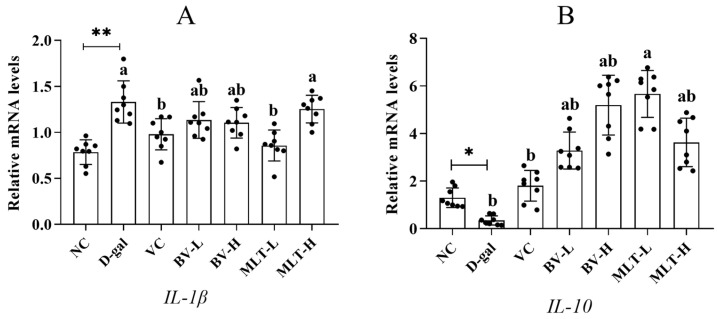
Effect of BV and MLT on the expression of *IL-1β* (**A**) and *IL-10* (**B**) in mice skin. NC, normal control; D-gal, D-galactose; VC, positive control; BV-L, low dose of bee venom (0.1 mg·kg^−1^); BV-H, high dose of bee venom (1 mg·kg^−1^); MLT-L, low dose of melittin (0.05 mg·kg^−1^); and MLT-H, high dose of melittin (0.5 mg·kg^−1^). ** indicates *p* < 0.01 vs. NC. Different letters indicate *p* < 0.05 between treatment groups.

The expression of IL-1β in the D-gal group was significantly elevated compared with that in the NC group (*p* < 0.01). After administration, the MLT-L and VC groups showed a significant decrease compared with the D-gal group (*p* < 0.05), and the rest of the groups showed decreasing trends; however, the differences were not significant (Figure 5A). The expression of IL-10 was significantly lower in the D-gal group than in the NC group (*p* < 0.05), whereas it was higher in the BV and MLT groups than in the D-gal group; however, only the MLT-L group showed a significant increase (*p* < 0.05; Figure 5B). This indicated that BV and MLT can regulate the expression of inflammatory factors in mouse skin to a certain extent and alleviate the skin inflammatory response.

### 2.4. Metabolomic Analysis with BV and MLT Treatment

Quality control (QC) samples were prepared by mixing the extracts of mouse skin samples, which were used to monitor the reproducibility of the samples under the same treatments. The high Pearson correlation between QC samples (r > 0.99) indicated the reliability of the data in this experiment; that is, the correlation between mouse skin samples was good, and the data quality was high. The metabolomics outputs have been submitted to the public repository of Metabolights (accession number: MTBLS11975, www.ebi.ac.uk/metabolights/MTBLS11975, accessed on 20 December 2024).

**Figure 6:**
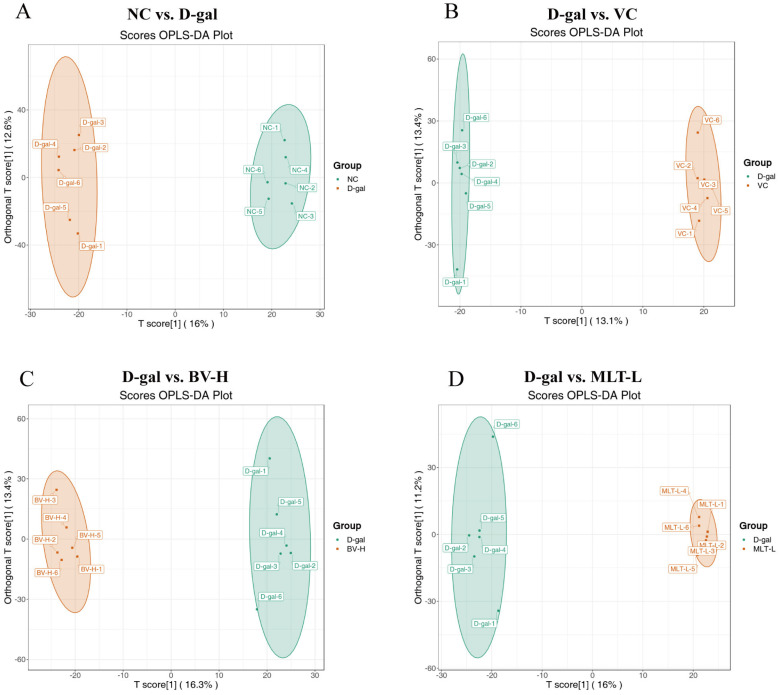
Comparison of orthogonal partial least squares discriminant analysis (OPLS-DA) score plots of mouse skin metabolism profiles from five groups. (**A**) NC vs. D-gal; (**B**) D-gal and VC; (**C**) D-gal vs. BV-H; (**D**) D-gal vs. MLT-L. NC, normal control; D-gal, D-galactose; VC, positive control; BV-H, high dose of bee venom; MLT-L, low dose of melittin.

To validate the orthogonal partial least squares discriminant analysis (OPLS-DA) model, 200 random permutation experiments were conducted on the data for this model, and the model was evaluated using R^2^ and Q^2^ values. The results were as follows: for the NC/D-gal group, R^2^Y = 0.993, Q^2^ = 0.663 (Figure 6A); for the D-gal/VC group, R^2^Y = 0.999, Q^2^ = 0.36 (Figure 6B); for the D-gal/BV-H group, R^2^Y = 0.992, Q^2^ = 0.636 (Figure 6C); and for the D-gal/MLT-L group, R^2^Y = 0.995, Q^2^ = 0.645 (Figure 6D). The OPLS-DA model was valid for all groups and provided a good explanation and prediction of differences between groups, except for the VC/D-gal group, for which the OPLS-DA model was poor.

**Figure 7:**
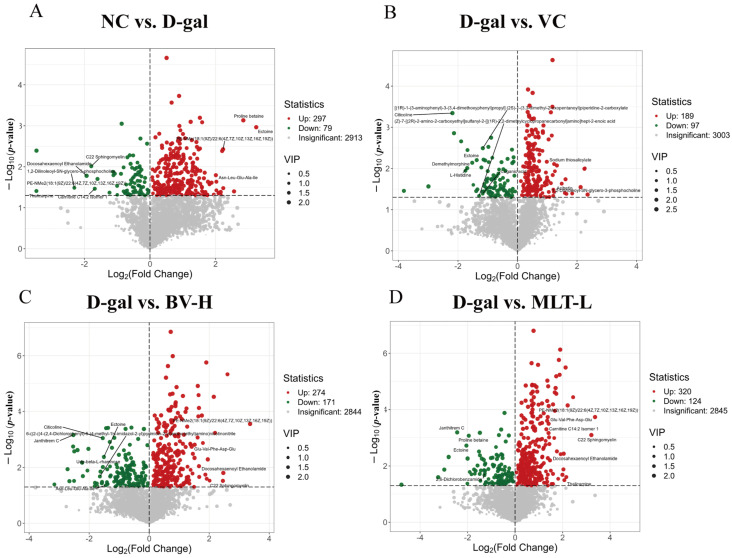
Volcano diagram of differential metabolites between different groups. (**A**–**D**) Volcano plots of differential metabolites between D-gal and NC groups, VC and D-gal groups, BV-H and D-gal groups, MLT-L and D-gal groups, respectively. The top 10 relevant metabolites ranked by log_2_FC-value are shown in each figure.

Based on the differential metabolite screening criteria, 376 differential metabolites were identified between the NC and D-gal groups, comprising 297 upregulated and 79 downregulated metabolites in the D-gal group (Figure 7A); 286 differential metabolites between the VC and D-gal groups, comprising 189 up-regulated and 97 downregulated metabolites in the VC group (Figure 7B); the BV-H and D-gal groups shared 445 metabolites, comprising 274 up-regulated and 171 downregulated metabolites in BV-H group (Figure 7C); and the MLT-L group and D-gal group shared 444 metabolites, comprising 320 upregulated metabolites and 124 downregulated metabolites in the MLT-L group (Figure 7D). The heatmaps for all samples and variable metabolites are shown in Figure S1.

**Figure 8:**
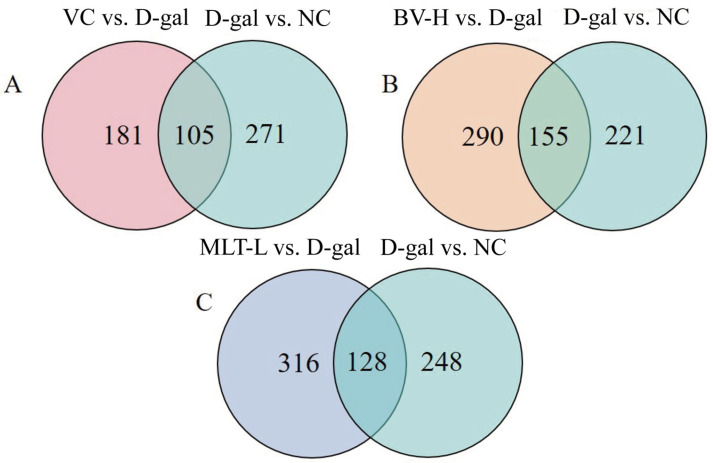
Venn diagram showing the number of metabolites shared among different groups. (**A**,**B**,**C**) represent the number of common differential metabolites in the VC, BV-H, and MLT-L groups shared by the NC and D-gal groups, respectively. NC, normal control; D-gal, D-galactose; VC, positive control; BV-H, high dose of bee venom; MLT-L, low dose of melittin.

Metabolites in common in each test group and the NC and D-gal groups were identified using Wayne’s analysis, of which 105 substances were shared by the VC, D-gal, and NC groups, and 155 and 128 substances were shared by the BV-H and MLT-L groups, respectively, with the NC and D-gal groups (Figure 8). Information regarding the metabolites of the aging model vs. NC groups and treatment vs. aging model groups is tabulated in Tables S1–S4. Further analysis revealed that 39, 56, and 35 differential metabolites were significantly regressed in the VC, BV-H, and MLT-L groups, respectively, compared with the D-gal group (Table S5).

### 2.5. KEGG Enrichment Analysis of Differential Metabolites

**Figure 9:**
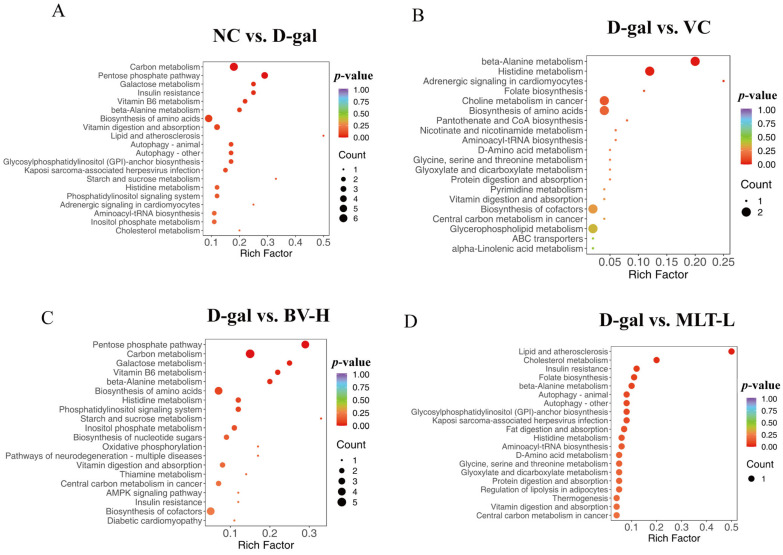
Differential metabolite pathway enrichment analysis bubble chart. (**A**–**D**) Enriched bubble plots of differential metabolite pathways between D-gal and NC groups, VC and D-gal groups, BV-H and D-gal groups, and MLT-L and D-gal groups, respectively.

The metabolic pathways of the various metabolites were analyzed, and the top 20 enriched metabolic pathways ranked by *p*-value are displayed in Figure 9. KEGG bubble plots show that the differential metabolites in the D-gal group were involved in carbon (*p* = 0.001), pentose phosphate (*p* = 0.002), and galactose (*p* = 0.0039), insulin resistance (*p* = 0.039), and VB6 (*p* = 0.049) metabolism pathways compared with those of the NC group (Figure 9A). The VC group was involved in β-alanine (*p* = 0.007), histidine (*p* = 0.018), and other metabolism pathways (Figure 9B); the BV-H group was involved in the phosphopentose (*p* = 0.001), carbon (*p* = 0.003), galactose (*p* = 0.023), vitamin B6 (*p* = 0.030), alanine (*p* = 0.036), and other metabolic pathways (Figure 9C); and the MLT-L group was involved in lipid, atherosclerosis (*p* = 0.016), cholesterol (*p* = 0.040), and other metabolism pathways (Figure 9D) compared with the D-gal group. Differential metabolites in the BV-H and MLT-L groups were collectively involved in β-alanine, histidine, vitamin digestion, and absorption, central carbon in cancer, and insulin resistance metabolic pathways.

## 3. Discussion

The structural integrity of the skin tissue is essential for its ability to perform a wide range of biological functions [13]. The skin shows obvious signs of aging with age, manifested as poor elasticity, weakened hydration, increased trans epidermal water loss, and decreased collagen content. Numerous studies have confirmed that the effect of D-gal induced aging is consistent with that of natural aging, supporting the application of the D-gal aging model [14,15,16]. Additionally, D-gal can lead to the thinning of the epidermal layer of mouse skin, decreased collagen fiber content, sparse arrangement, and a decreased number of skin appendages [17]. Our study confirmed these findings through HE and Masson staining, which were used to observe changes in the skin of D-gal aging model mice. The epidermis and dermis of the skin were thickened, the number of subcutaneous fat cells was reduced, and collagen fibers were more abundant and well-arranged after treatment with BV and MLT, suggesting that these two treatments delay the aging of mouse skin by resisting external harmful substances.

Collagen, is an extracellular protein, is the most abundant protein in the body, with human skin consisting of 70% collagen [18]. It plays a vital role in the structure and function of the skin, and aging leads to decreased collagen levels. The collagen superfamily comprises 28 members. Type I collagen determines skin contour, whereas type III collagen determines skin elasticity and fineness. High doses, as well as both high and low doses of MLT, significantly enhanced the expression of *Col1a1* and *Col3a1* in aging mice. The low dose of MLT was more effective. Hyp is a characteristic amino acid in skin collagen that promotes the growth of skin fibroblasts [19]. HA is a mucopolysaccharide with specific water-retaining properties that promotes wound healing, improves skin nutrient metabolism and moisturization, and has anti-aging properties [20]. In the present study, we found that both BV and MLT significantly increased the levels of HA and Hyp in senescent mouse skin sections, with BV-H exhibiting more pronounced anti-aging activity. BV-H and MLT-L had greater effects on the expression of anti-aging–related proteins. The reason for this dose-dependent effect remains unknown.

Increased levels of inflammatory cytokines also contribute to accelerated aging, as evidenced by elevated levels of inflammatory response markers such as TNF-α and IL-1β [21]. IL-1 initiates the inflammatory response, with pro-inflammatory effects, and its activity is mainly expressed by IL-1β. IL-10 is the most potent cytokine in the body and is commonly used to evaluate the degree of inflammatory senescence [22]. In this study, we demonstrated that BV and MLT down-regulate the expression of IL-1β and up-regulate that of IL-10 in senescent mouse skin sections., The effects of the BV-H and MLT-L groups were better, which were close to or even higher than those of the VC group, which indicated that both BV and MLT had good anti-inflammatory effects. In a previous study, we showed that BV has dose-dependent effects on both pro- and anti-inflammatory factors, which is consistent with the results of the current study [23].

The skin aging process is affected by circulating metabolites that are involved in a variety of cellular processes, including cellular organization, post-translational modifications, and epigenetic changes [24]. Furthermore, these metabolites are chemical reaction products of the physiological functions of the body [25]; therefore, studying mouse skin aging metabolites is helpful for understanding the aging mechanism further. Metabolomic analysis revealed differential metabolites that regulate disorders caused by D-gal. These substances included metabolites such as carbohydrates, amino acids, and glycerophospholipids. The number of differential metabolites was greater in the BV-H group than in the MLT-L and VC groups, including pyridoxamine heterocyclic compounds, D-fructose-6-phosphate, Asn-Leu-Glu-Ala-Ile, D-tagatose 6-phosphate, pyrophosphate, and and UDP-beta-L-rhamnose, suggesting that the anti-aging effect of BV is superior to that of MLT.

Glucose, protein, and lipid metabolism affect skin aging, and glucose and galactose levels are directly related to skin aging [26]. Disturbed glucose metabolism affects the metabolism of proteins and lipids, reduces the ability of cells to scavenge free radicals, and covalently binds proteins in the body, leading to severe cellular damage, such as decreased lipid peroxidation and organelle function [27]. D-fructose-6-phosphate and D-tagatose-6-phosphate are involved in galactose and carbon metabolism pathways. In this study, the levels of these metabolites were decreased in the BV-H group relative to those in the D-gal group, effectively alleviating D-gal-induced disturbance of glucose metabolism. Additionally, the pentose phosphate metabolic pathway of glucose metabolism is highly active in the skin and plays an essential role in the growth and repair of skin tissue cells [28]; pentose phosphate metabolism is reduced in aging skin cells [29]. In the KEGG enrichment analysis, we found that 6-phosphogluconate content was higher in the BV-H group than in the D-gal group, and the D-erythrose-4-phosphate, 7-phosphogluconoheptulose, and 6-gluconosyl-6-phospho-d-lactone content was decreased. All these metabolites are involved in the pentose phosphate pathway, suggesting that BV and MLT enable skin tissues to resist the metabolic disturbances induced by D-gal.

The skin is rich in fibrous proteins such as keratin, collagen, and elastin, and amino acids are the basic building blocks of proteins. Studies have shown that amino acid metabolism changes with age. With increasing age, the amino acid conversion rate in the body decreases significantly, and protein anabolism decreases [30]. We found that L-histidine, tyrosine-arginine-isoleucine-glutamate, and L-arginyl-L-prolyl-glycine levels were significantly elevated in the D-gal group, suggesting that D-gal disturbed the balance of amino acid metabolism in the mouse skin sections [31]. After treatment with BV and MLT, the levels of L-histidine decreased, indicating that the BV-H and MLT-L might accelerate the absorption and use of L-histidine, thereby decelerating skin aging.

Reduced glycerolipid metabolism during aging may severely affect the lipid layer of the stratum corneum epidermal barrier and lead to impaired barrier function in aged skin [32]. Carnitine is essential in fatty acid metabolism, and fatty acids must be facilitated by carnitine to enter the mitochondria to undergo β-oxidation. In this experiment, the carnitine content decreased significantly after D-gal treatment, which in turn inhibited the oxidation of long-chain fatty acids, consistent with the metabolomics results for procyanidin B2 in aged mice [33]. Additionally, carnitine content was found to rebound after MLT-L treatment. The carnitine content was also elevated after treatment in the BV-H group. Glycerol 1-myristate was also decreased in the MLT-L group compared to the D-gal group; this metabolite is involved in the pathways of lipid and atherosclerosis, insulin resistance, cholesterol metabolism, and glycerolipid metabolism. These results suggest that BV and MLT improve abnormal fatty acid metabolism and promote lipid synthesis, thus delaying the aging of mouse skin.

Based on a non-targeted metabolomics study of mouse skin, BV alleviated D-gal-induced skin aging in mice by participating in glucose and amino acid metabolism. In contrast, MLT appears to alleviate D-gal-induced metabolic disorders by participating in the lipid, atherosclerosis, and cholesterol metabolism pathways. Compared to MLT, BV has a more complex composition, which may lead to more modifiable differential metabolites. Further exploration of the specific mechanisms of BV and MLT in anti-aging is required.

In this study, via the visualization the internal structure of the skin of mice subjected to D-gal-induced skin aging, determination of anti-aging factors, and analysis of skin metabolites, the mechanism of the anti-aging effect of BV was explored. However, these indicators are superficial, and their relationship with the specific anti-aging mechanism needs to be further explored. Additionally, BV and its isolated component need to be more carefully analyzed in terms of dose-dependent effects in skin anti-aging applications.

## 4. Materials and Methods

### 4.1. Experimental Animals

Specific pathogen-free male Kunming mice, aged 6 weeks, were purchased from SBF Biotechnology Co., Ltd. (production license no.: SCXK 2019–0010; Beijing, China). All procedures strictly followed the international experimental rules for animals and internationally recognized ethical laboratory animal use and care principles. The Ethics Committee of Shanxi Agricultural University, China, approved the experimental procedures (Approval No. SXAU-EAW-2023M.HJ.003014220). The mice were kept in a well-ventilated facility, with an environmental temperature of 24 ± 2 °C, relative humidity of 40–70%, a 12/12 h light/dark cycle, and food and water were freely available.

BV (purity: 90%) was purchased from Anhui Jingfeng Bee Venom Biotechnology Co., Ltd. (Maanshan, Anhui, China). MLT (purity: 98.9%, LC) was purchased from Yuantai Biotechnology Co., Ltd. (Nanjing, China). VC (purity: 99.0%, AR) was purchased from Solarbio technology Co., Ltd. (Beijing, China).

### 4.2. Experimental Design in Mice

In total, 56 mice were randomly separated into 7 groups (*n* = 8 in each group) after 1 week of adaptive feeding: the normal control group (NC: 0.9% saline), the aging model group (D-gal: 500 mg·kg^−1^), the VC group (positive control; VC: 100 mg·kg^−1^), low-dose BV group (BV-L: 0.1 mg·kg^−1^), high-dose BV group (BV-H: 1 mg·kg^−1^), low-dose MLT group (MLT-L: 0.05 mg·kg^−^1), and high-dose MLT group (MLT-H: 0.5 mg·kg^−1^). Agents were prepared in saline. The NC and D-gal groups received injections once a day via the scruff of the neck. The other five treatment groups also received D-gal every day. VC was administered via gavage, and BV and MLT were injected once every 2 days. The experiment lasted 7 weeks.

### 4.3. Morphological Observation of Skin

After the mice were euthanized, the hair on the back of the neck was scraped off using scissors and a scalpel, and the skin tissue (approximately 1 × 1 cm^2^) was removed from the back, fixed in 4% paraformaldehyde solution, and subjected to HE and Masson trichrome staining after dehydration, embedding, sectioning, and deparaffinization [34]. Skin tissue morphology was observed for each group using a panoramic scanner (3D HISTECH, Budapest, Hungary).

### 4.4. Determination of the Content of Hyaluronic Acid and Hydroxyproline

Skin tissue (100 mg) from the back of each mouse was homogenized, and 0.9 mL of saline was added to the homogenate. The concentration of Hyp and HA in the supernatant of 10% skin tissue homogenate was detected using ELISA based on the specific binding of antigens and antibodies. The assay was performed as per the manufacturer’s instructions (Jining Biotechnology Co., Ltd., Shanghai, China).

### 4.5. Measurement of the Expression Levels of Collagen and Inflammatory Factors

Skin tissue (100 mg) was collected from each mouse and pulverized using liquid nitrogen grinding. Total RNA was isolated using TRIzol reagent (Invitrogen, Carlsbad, CA, USA) and the PrimeScript II 1st Strand cDNA Synthesis Kit (Takara Bio, Kusatsu, Japan) according to the manufacturers’ instructions. The qRT-PCR for testing the mRNA expression levels of *Col1a1*, *Col1a3*, *IL-1β*, and *IL-10* was performed using PrimeScript™ RT reagent Kit with gDNA Eraser (Takara Bio, Kusatsu, Japan). Each sample was analyzed in triplicate to ensure data reproducibility. The relative gene expression value was calculated using the comparative 2^−ΔΔCt^ method [35]. The primer sequences used for amplification are listed in Table S6.

### 4.6. Metabolomics Analysis

We selected the BV-H and MLT-L groups for metabolomic analysis. Overall, 30 skin samples (NC, D-gal, VC, BV, and MLT groups, n = 6 in each group) were analyzed. The skin tissues (20 mg of each sample) were homogenized and centrifuged, followed by the addition of a 70% methanol–water internal standard (400 μL). Subsequently, the supernatant was collected after shaking, standing for 30 min at −20 °C, and centrifugation. The final supernatant was transferred to the corresponding injection vials for metabolomic analysis.

Sample extracts were analyzed using liquid chromatography electrospray ionization tandem mass spectrometry (LC-ESI-MS/MS) system (Shimadzu, Jingdu, Japan). The precise conditions for chromatography were determined as described previously [36]. The chromatographic and MS parameters are listed in Tables S7 and S8.

The analytical conditions were as follows: column, Waters ACQUITY UPLC HSS T3 C18 (1.8 µm, 2.1 mm × 100 mm); column temperature, 40 °C; flow rate, 0.4 mL/min; injection volume, 4 μL; and solvent system, water (0.1% formic acid)/acetonitrile (0.1% formic acid).

### 4.7. Metabolomics Data Processing

The metabolic characteristics of QC samples with a relative standard deviation greater than 30% and disrupted signals in the blanks were removed to avoid interference. QC samples were prepared by mixing extracts of samples to be tested. For the remaining qualified data, OPLS-DA was conducted and volcano plots were constructed using SIMCA 14.1 (Umetrics, Umea, Sweden). The data were log-transformed and mean-centered before OPLS-DA. A permutation test (200 permutations) was performed to avoid overfitting. The metabolites were identified using eXtensible Computational Mass Spectrometry. The following databases were used for the identification of the metabolites: KEGG, HMDB, CHEBI, and MetaboAnalyst.

### 4.8. Statistical Analysis

Statistical analyses were performed using IBM SPSS 26.0. Normally and approximately normally distributed data are expressed as mean ± standard error. Student’s *t*-tests were used to compare the NC and D-gal groups, and one-way analysis of variance (ANOVA) was used to compare the D-gal group and each experimental group. GraphPad Prism 8.0 was used to plot results.

To identify metabolic features that set the control group apart from the treatment groups, the criteria were considered as FDR (false discovery rate) adjusted *p* < 0.05, VIP (variable importance in projection) ≥ 1, and absolute Log_2_FC (fold change) ≥1.

## 5. Conclusions

Our findings indicate that BV and MLT enhance the collagen fiber content. They increase the protein levels of Hyp and HA, increase the gene expression levels of *Col1a1* and *Col3a1,* and regulate the gene expression of *IL-1β* and *IL-10*. BV-H and MLT-L have stronger effects on the recovery of collagen content and alleviation of inflammation. BV-H alleviates D-gal-induced skin aging by regulating the pentose phosphate pathway, carbon metabolism, galactose metabolism, and β-alanine metabolism. MLT-L alleviates D-gal-induced skin metabolic disorders by regulating lipid, atherosclerosis, and cholesterol metabolism pathways.

BV and MLT may alleviate skin aging in mice by inhibiting inflammation; promoting collagen synthesis, and regulating glucose, protein, and lipid metabolism. These findings provide a scientific basis for the use of BV in delaying skin aging. Future studies should consider delving into mechanisms underlying the side effects of formulations containing BV; this is because lytic peptides present in BV have been shown to yield adverse reactions in some people.

## Data Availability

The data presented in this study are available upon request from the corresponding author.

## Supplementary Materials

The following supporting information can be downloaded at: https://www.mdpi.com/article/10.3390/ijms26020742/s1.
