# Supplementary material for: Study on the Effect of Bee Venom and Its Main Component Melittin in Delaying Skin Aging in Mice
Source: Int J Mol Sci. 2025 Jan 16;26(2):742. doi: 10.3390/ijms26020742 (PMC11766253; doi:10.3390/ijms26020742)
Supplement: Supplementary file 1 [file ijms-26-00742-s001.zip › Table S5-S8.pdf]

Table S5 Different metabolites with BV and MLT treatment

Material ID	Compounds	Molecular formula	D-gal/ NC	VC/ D-gal	BV-H/ D-gal	Mlt-L/ D-gal
MW0057055	1,2-Dilinoleoyl-SN-glycero-3-phosphocholine	C44H80NO8P	↓	↑	—	—
MW0169549	Niacinamide	C6H6N2O	↑	↓	↓	—
MW0106578	Ectoine	C6H10N2O2	↑	↓	↓	↓
MW0169728	Pyridoxamine Heterocyclic compounds	C8H12N2O2	↑	—	↓	—
MW0055261	C22 Sphingomyelin	C45H91N2O6P	↓	—	↑	↑
MW0114686	Tartaric acid	C4H6O6	↑	↓	↓	↓
MW0007422	Isoproterenol	C11H17NO3	↓	↑	—	—
MW0151906	JanthitremC	C37H47NO4	↑	↓	↓	↓
MW0107334	L-Histidine	C6H9N3O2	↑	↓	↓	↓
MW0011245	(3E,5Z)-3,5-Octadien-1-ol	C8H14O	↑	—	↓	↓
MEDP1847	5-Hydroxymethyl-2-furancarboxaldehyde	C6H6O3	↑	↓	↓	↓
MEDP1136	D-Fructose-6-phosphate	C6H13O9P	↑	—	↓	—
MEDP1610	Proline betaine	C7H13NO2	↑	—	↓	↓
MW0103666	Telbivudine	C10H14N2O5	↑	↓	↓	↓
MW0111694	1,2-Ethanediol	C2H6O2	↓	—	↑	—
MW0168478	2,6-Dichlorobenzamide	C7H5Cl2NO	↑	—	↓	↓
MW0111798	1-deoxy-D-xylulose 5-phosphate	C5H11O7P	↑	—	↓	—
MW0114481	L-fucopyranose 1-phosphate	C6H13O8P	↑	—	↓	—
MW0111016	Docosahexaenoyl Ethanolamide	C24H37NO2	↓	—	↑	↑
MW0062178	Pristanic acid Terpenoids	C19H38O2	↓	—	↑	—
MW0142235	2-Amino-6-methoxypurine	C6H7N5O	↑	—	↓	—
MW0145807	Asn-Leu-Glu-Ala-Ile	C24H42N6O9	↑	—	↓	—
MW0108413	N-Phenethylacetamide	C10H13NO	↓	—	↑	↑
MW0119751	6-((2-((4-(2,4-Dichlorophenyl)-5-(4-methyl-1H-imidazol-2-yl)pyrimidin-2-yl)amino)ethyl)amino)nicotinonitrile	C22H18Cl2N8	↑	—	↓	—
MW0114373	D-tagatose 6-phosphate	C6H13O9P	↑	—	↓	—
MW0161604	Fructose-6-Phosphate (closed form)	C6H13O9P	↑	—	↓	—
MW0060385	PE-NMe2(18:1(9Z)/22:6(4Z,7Z,10Z,13Z,16Z,19Z))	C47H80NO8P	↓	—	↑	↑
MW0161532	Dihydroxy-2-butanone-4-phosphate	C4H9O6P	↑	—	↓	—
MW0158010	trans-Zeatinriboside diphosphate	C15H23N5O11P2	↑	—	↓	—
MW0104888	3-(Imidazol-4-yl)-2-oxopropyl dihydrogen phosphate	C6H9N2O5P	↑	↓	↓	—
MW0005838	6-Phosphogluconic acid	C6H13O10P	↓	—	↑	—
MEDN1302	1D-myo-inositol 1,4-bisphosphate	C6H14O12P2	↑	—	↓	—
MEDP1055	DL-Glyceraldehyde 3-Phosphate	C3H7O6P	↑	—	↓	—
MEDL02772	D-Erythrose 4-phosphate	C4H9O7P	↑	—	↓	—
MW0106488	2,3-Diaminopropionic acid	C3H8N2O2	↑	↓	—	—
MW0151726	1-O-Indol-3-ylacetyl-beta-D-glucose	C16H19NO7	↓	↑	↑	—
MW0150216	Glu-Val-Phe-Asp-Glu	C28H39N5O12	↓	↑	↑	↑
MW0158377	Tyr-Arg-Ile-Glu	C26H41N7O8	↑	↓		—
MW0145479	Arg-Pro-Gly	C13H24N6O4	↑	↓	—	—
MW0155767	Pro-Ala-Leu	C14H25N3O4	↓	↑	—	—
MW0129640	1,26-Hexacosanediol diferulate	C46H70O8	↑	↓	—	—
MEDP1592	Citicoline	C14H26N4O11P2	↑	↓	↓	—
MW0016134	Betamethasone acetate	C24H31FO6	↑	↓	—	↓
MW0152783	L-(-)-Sorbose	C6H12O6	↓	↑	↑	—
MEDN1780	Pseudolaric acid B	C23H28O8	↑	↓	—	—
FDATN00366	Phenylephrine hydrochloride	C9H14ClNO2	↓	↑	↑	↑
MW0127079	Methylarsonate	CH3AsO3-2	↑	↓	↓	—
MW0052386	Dihydrouracil	C4H6N2O2	↑	↓	↓	—
MW0126146	Pinidine	C9H17N	↓	↑	↑	—
MW0015892	Arbutin	C12H16O7	↑	—	↓	—
MW0105990	gold(1+);(2S,3R,4S,5R,6R)-3,4,5-triacetyloxy-6-(acetyloxymethyl)oxane-2-thiolate;triethylphosphane	C20H34AuO9PS	↓	—	↑	↑
MW0115265	Sedoheptulose 7-phosphate	C7H15O10P	↑	—	↓	—
MW0114218	D-Glucono-1,5-lactone 6-phosphate	C6H11O9P	↑	—	↓	—
MEDN0659	Pyrophosphate	H4O7P2	↑	—	↓	—
MW0154511	Nigrifactin	C12H17N	↓	↑	↑	↑
MW0054403	Loganic acid	C16H24O10	↑	↓	—	—

MW0140737	[(1R)-1-(3-aminophenyl)-3-(3,4-dimethoxyphenyl)propyl] (2S)-1-(3,3-dimethyl-2-oxopentanoyl)piperidine-2-carboxylate	C30H40N2O6	↑	↓	↓	—
MW0103690	Udp-beta-L-rhamnose	C15H24N2O16P2	↑	—	↓	—
MEDP1057	d-Myo-inositol 4-monophosphate ammonium salt	C6H13O9P	↑	—	↓	—
MW0115384	trans-1,2-Dihydrobenzene-1,2-diol	C6H8O2	↑	—	↓	↓
MW0106194	Cyanidin 3-gentiobioside	C27H31O16+	↓	—	↑	↑
MW0115724	(-)-3-Cyanomethyl-3-hydroxy-1H-indol-2(3H)-one	C10H8N2O2	↑	↓	↓	—
MW0157113	Sodium thiosalicylate	C7H5NaO2S	↓	↑	—	—
MW0115419	Triclabendazole	C14H9Cl3N2OS	↑	↓	↓	—
MW0105209	4-Fluoro-L-phenylalanine	C9H10FNO2	↑	↓	—	—
MW0143248	4-Amino-4-deoxychorismic acid	C10H11NO5	↑	↓	↓	↓
MW0160218	Demethylmorphine	C16H17NO3	↑	↓	—	—
MW0153410	m-Benzenesulfonium diazonium chloride	C6H5ClN2O3S	↑	↓	↓	—
MW0115674	Chloroacetyl chloride	C2H2Cl2O	↓	—	↑	—
MW0009940	Toloxatone	C11H13NO3	↓	↑	—	—
MW0194144	amino-2-carboxyethyl]sulfanyl-2-[[(1R)-2,2-dimethylcyclopropanecarbonyl]amino]hept-2-enoic acid	C16H26N2O5S	↑	↓	—	—
MW0005966	Acitretin	C21H26O3	↓	↑	—	—
MEDP1407	Carnitine C14:2	C21H37NO4	↓	—	—	↑
MW0157470	Thalicarpine	C41H48N2O8	↓	—	—	↑
MEDP1406	Carnitine C14:1	C21H39NO4	↓	—	—	↑
MW0143550	5alpha-Cholane	C24H42	↓	—	—	↑
MW0169676	Pinolenic acid	C18H30O2	↓	—	—	↑
MEDP1521	Carnitine C14:2 Isomer 1	C21H37NO4	↓	—	—	↑
MW0006939	Drinabant	C23H20Cl2F2N2O2S	↑	—	—	↓
MW0053713	Glycerol 1-myristate	C17H34O4	↑	—	—	↓
MW0140152	13-Hydroxydocosa-4,7,10,14,16,19-hexaenoic acid	C22H32O3	↑	—	—	↓
MW0006426	Buclizine	C28H33ClN2	↑	—	—	↓
MW0124192	Furmecyclox	C14H21NO3	↓	—	—	↑
MW0105859	Asp-Ile	C10H18N2O5	↓	—	—	↑
MW0123283	Cephaloglycin	C18H19N3O6S	↑	—	—	↓
MW0158678	Tyr-Abu-OH	C19H20N2O7	↑	↓	—	—

Note：—represents insignificant differences in substances between groups

Table S6 Primer information for qRT-PCR

Primer		Primer sequences（5′-3′）
β-actin	F	TACCCAGGCATTGCTGACAG
	R	CGGACTCATCGTACTCCTGC
Col1a1	F	GACAGGCGAACAAGGTGACAGAG
	R	CAGGAGAACCAGGAGAACCAGGAG
Col3a1	F	ACGAGGTGACAAAGGTGAAACTGG
	R	AGAACCTGGAGGACCTGGATTGC
IL-1β	F	CACTACAGGCTCCGAGATGAACAAC
	R	TGTCGTTGCTTGGTTCTCCTTGTAC
IL-10	F	CTGGACAACATACTGCTAACCGACTC
	R	ACTGGATCATTTCCGATAAGGCTTGG

Table S7 Gradient conditions for mobile phase of T3 chromatographic column

Time (min)	A (%)	B (%)
0.0	95	5
2.0	80	20
5.0	40	60
6.0	1	99
7.5	1	99
7.6	95	5
10.0	95	5

Table S8 AB TripleTOF 6600 mass spectrometry conditions

Name	ESI+	ESI-
Duration (min)	10	10
IonSpray Voltage (V)	5000	-4000
Temperature (°C)	550	450
Ion Source Gas1 (psi)	50	50
Ion Source Gas2 (psi)	60	60
Curtain Gas (psi)	35	35
Declustering Potential (V)	60	-60
MS1 Collision Energy (V)	10	-10
MS2 Collision Energy (V)	30	-30
Collision Energy Spread (V)	15	15
MS1 TOF Masses (Da)	50~1000	50~1000
MS2 TOF Masses (Da)	25~1000	25~1000
MS1 Accumulation time (s)	0.2 s	0.2 s
MS2 Accumulation time (s)	0.04 s	0.04 s
Candidate ions	18	18
