# Supplementary figures and images for: Study on the Effect of Bee Venom and Its Main Component Melittin in Delaying Skin Aging in Mice
Source: Int J Mol Sci. 2025 Jan 16;26(2):742. doi: 10.3390/ijms26020742 (PMC11766253; doi:10.3390/ijms26020742)

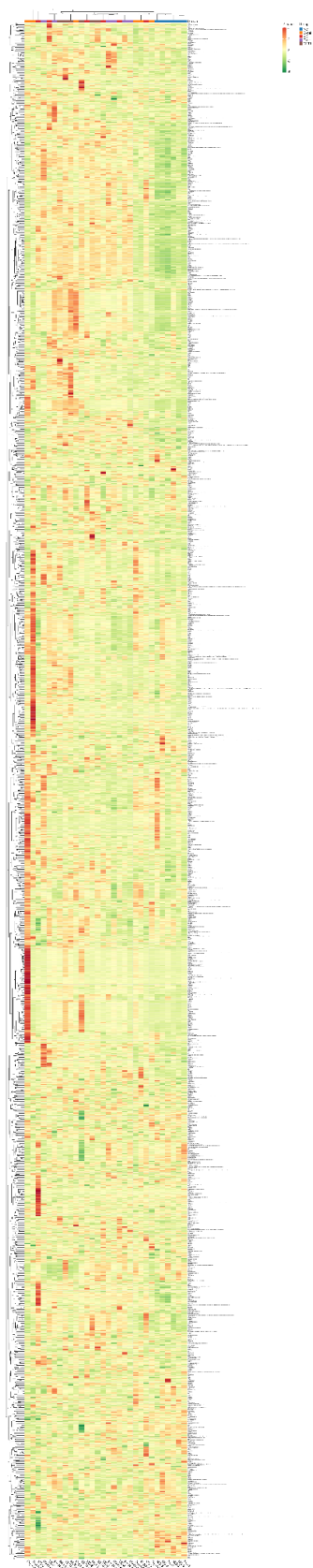

Figure S1: The heatmaps for all samples and variable metabolites.

Supplement: Supplementary file 1 [file ijms-26-00742-s001.zip › Figure S1.pdf]
